# Impact of smoking cessation on survival and treatment tolerability in advanced non-small cell lung cancer: a single-center experience from Morocco

**DOI:** 10.3332/ecancer.2026.2110

**Published:** 2026-04-09

**Authors:** Intissar Belrhali, Ibrahim El Ghissassi, Boutaina Cherkaoui, Oumaima Lamsyah, Khaoula Ouchen, Soufiane Bel Rhali, Saoussane Kharmoum, Sarah Naciri, Hanane Inrhaoun, Siham Lkhoyaali, Salma Najm, Hind Mrabti, Hassan Errihani

**Affiliations:** 1 Department of Medical Oncology, National Institute of Oncology, Rabat 10000, Morocco; 2 Department of Immunology, MD Anderson Cancer Center, Houston, TX 77030, USA; 3 Department of Molecular Virology and Microbiology, Baylor College of Medicine, Houston, TX 77030, USA; 4 Department of Medical Oncology, Regional Hospital Center, Tanger 90000, Morocco

**Keywords:** non-small cell lung cancer, smoking cessation, overall survival, treatment tolerability, haematologic toxicity, Morocco

## Abstract

**Background:**

Smoking cessation following a lung cancer diagnosis presents a dual challenge, as patients must cope with both the emotional burden of cancer and nicotine dependence. This study aimed to evaluate the impact of smoking cessation on overall survival (OS) and treatment tolerability in patients with locally advanced or metastatic non-small cell lung cancer (NSCLC).

**Methods:**

This retrospective cohort study included patients with stage III–IV NSCLC treated at the National Institute of Oncology, Rabat, Morocco, between January 2022 and December 2023. Patients were classified as ex-smokers (n = 60), who quit smoking at diagnosis or active smokers (n = 40), who continued smoking thereafter. Smoking cessation support was individualised and included medical counselling, psychological support, nicotine replacement therapy and referral to specialised cessation consultations. Outcomes assessed included treatment response, adverse events and OS.M

**Results:**

Among 100 patients (mean age 69 ± 9 years), there were no significant differences between groups in disease stage (p = 0.70) or histology (p = 0.63). Treatment intolerance occurred in 55% of patients, with adverse events more frequent in active smokers (39%) than in ex-smokers (16%). Haematologic toxicity, particularly neutropenia, was significantly higher in active smokers (p < 0.001). Notably, patients who quit smoking during treatment experienced improved treatment tolerability. Median OS was 10 months overall, with longer survival in ex-smokers (11 months) compared with active smokers (7 months) (log-rank p < 0.001).

**Conclusion:**

Smoking cessation at diagnosis is associated with reduced treatment-related toxicity and improved survival in advanced NSCLC, supporting its role as an independent prognostic factor and a priority in clinical management.

## Introduction

Lung cancer remains the leading cause of cancer-related mortality worldwide, with an estimated 2.21 million new cases and 1.8 million deaths reported in 2020 [1]. In Morocco, approximately 8,825 new cases were diagnosed that year [1]. Non-small cell lung cancer (NSCLC) accounts for more than 80% of all lung cancer cases [1]. Despite advances in diagnostic techniques and therapeutic strategies, prognosis remains poor, with a global 5-year overall survival (OS) rate of only 26.7% across all stages [2].

The burden of lung cancer is strongly linked to tobacco consumption, particularly in industrialised nations where smoking prevalence peaked in the mid-20th century. Seminal epidemiological studies by Doll and Hill [3] and reports from the U.

S. Surgeon General [4] firmly established smoking as the primary etiologic factor. While tobacco use has declined in many high-income countries, prevalence is increasing in parts of Asia and Africa, contributing to a rising cancer burden [5].

Although only a fraction of smokers develop lung cancer, an estimated 80%–90% of cases in the United States are directly attributable to smoking [6]. The economic impact is also considerable: in 2008, the global cost of lung cancer was estimated at USD 180 billion [6], with treatment expenses in the United States exceeding USD 45,000 per patient [7]. Tobacco smoke contains more than 7,000 chemicals, including at least 69 known carcinogens [8].

Given this high mortality and substantial economic burden, identifying modifiable prognostic factors in NSCLC is a public health priority. One such factor is smoking behaviour after diagnosis. This study aimed to assess the impact of smoking cessation versus continued smoking on treatment tolerability and survival outcomes in patients with locally advanced or metastatic NSCLC.

## Materials and methods

This retrospective cohort study was conducted in the Department of Medical Oncology, National Institute of Oncology (INO), Rabat, Morocco. Medical records of patients diagnosed with locally advanced or metastatic NSCLC between January 2022 and December 2023 were reviewed.

### Eligibility criteria

Patients were eligible if they were aged ≥18 years, had histologically confirmed NSCLC, and reported active smoking at the time of diagnosis. Exclusion criteria included prior smoking cessation before diagnosis, never-smoker status, incomplete medical records, history of other active malignancies or enrollment in clinical trials involving investigational agents during the study period.

### Study groups

Patients were classified into two groups according to smoking status after diagnosis:

**Ex-smokers:** patients who quit smoking at diagnosis (*n* = 60).

**Active smokers:** patients who continued smoking after diagnosis (*n* = 40).

### Smoking cessation support

All patients who were active smokers at diagnosis were offered individualised smoking cessation support. Interventions included medical counselling by the treating oncologist, psychological support, encouragement of family involvement, nicotine replacement therapy (patches and/or gum) and referral to specialised smoking cessation consultations when available.

### Data collection

Data were collected using a standardised form and included: demographic characteristics (age, sex), history of other toxic habits (alcohol, cannabis), smoking status at diagnosis and during treatment, performance status (World Health Organization (WHO) scale), tumour histology and Tumour, Node, Metastasis (TNM) stage, molecular profile (epidermal growth factor receptor (EGFR), anaplastic lymphoma kinase (ALK), programmed death-ligand 1 (PD-L1)), metastatic sites, treatment regimens (type, number of cycles), clinical response, treatment-related toxicities and survival outcomes.

### Statistical analysis

Descriptive statistics were used to summarise baseline characteristics. Categorical variables were expressed as frequencies and percentages, and continuous variables as means or medians. OS was estimated using the Kaplan–Meier method, and survival curves were compared using the log-rank test. A *p*-value < 0.05 was considered statistically significant. Analyses were performed using SPSS version 24.0 (IBM Corp., Armonk, NY, USA).

### Ethical considerations

This study was approved by the Ethics Committee of the National Institute of Oncology, Rabat, Morocco, and conducted in accordance with the principles of the Declaration of Helsinki. All data were anonymised; individual patient consent was not required according to institutional policy.

## Results

### Baseline characteristics

Comorbidities were present in 11% of patients, including diabetes, hypertension and asthma. Regarding toxic habits, 12% were chronic alcohol users and 20% reported concurrent cannabis use.

At diagnosis, 75% of patients had metastatic disease. Group 1 (ex-smokers) included 60 patients, while Group 2 (active smokers) included 40 patients. A WHO performance status of 1 was observed in 54% of patients in Group 1 compared with 31% in Group 2.

Histologically, 76% of tumours were adenocarcinomas, 18% squamous cell carcinomas and 6% large-cell neuroendocrine carcinomas. Molecular alterations were detected in 38% of patients, including EGFR mutations (3%), ALK rearrangements (1%) and high PD-L1 expression (>50%) in 22%.

### Sites of metastasis

The most frequent metastatic sites were lymph nodes (75%), lungs (43%), pleura (32%), bones (27%), adrenal glands (19%), brain (13%) and liver (9%).

### Treatment modalities

Combination therapy consisting of chemotherapy and targeted agents, such as bevacizumab, was administered to 49% of patients. Chemotherapy alone was given in 39% of cases, while 8% underwent concurrent chemoradiotherapy (CRT). Among CRT recipients, 6% experienced disease relapse, 5% of whom were in Group 2.

### Tumour response

Following first-line chemotherapy, disease control – defined as complete response, partial response or stable disease – was achieved in 48 patients from Group 1, with a median duration of 5 months, which was significantly longer than in Group 2 (*p* = 0.02) (Table 1).

### Treatment-related toxicity

Overall, 55% of patients experienced treatment-related toxicities. Adverse events were significantly more frequent in Group 2 (active smokers) (39%) compared with Group 1 (ex-smokers) (16%). Haematologic toxicity was the most common, with neutropenia significantly more prevalent among active smokers (*p* < 0.001). Non-haematologic toxicities – including nausea, mucositis and fatigue – were less frequent and did not differ significantly between groups.

Treatment interruptions or dose reductions due to toxicity occurred more often in active smokers, although this difference did not reach statistical significance.

### Smoking cessation during treatment

Notably, several patients who were active smokers at baseline successfully quit smoking during the course of treatment. Clinically, smoking cessation during therapy was associated with improved treatment tolerability, including fewer treatment-related toxicities and less frequent treatment interruptions.

### Survival outcomes

**Figure 1.**
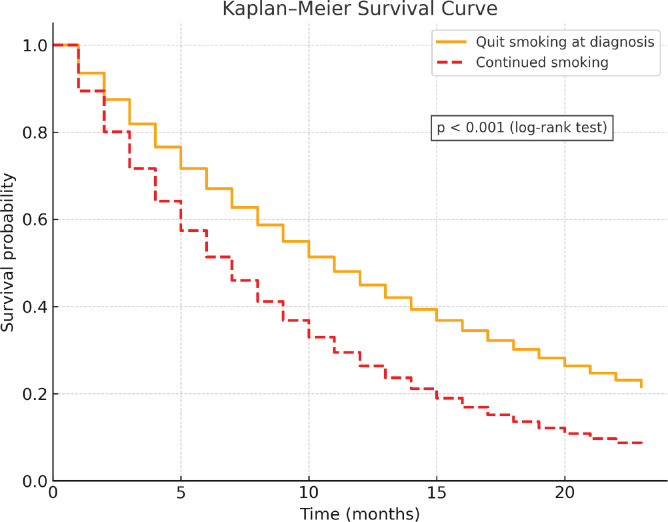
Kaplan–Meier survival curve comparing OS in ex-smokers and active smokers. The solid orange line represents patients who quit smoking at diagnosis, while the dashed red line represents those who continued smoking (p < 0.001, log-rank test).

The median OS for the entire cohort was 10 months. Patients who quit smoking at diagnosis had a median OS of 11 months, compared with 7 months in those who continued to smoke. Kaplan–Meier analysis demonstrated a statistically significant survival advantage for ex-smokers (*p* < 0.001, log-rank test) (Figure 1). At all time points, the probability of survival was consistently higher in patients who ceased smoking following diagnosis.

## Discussion

Lung cancer remains one of the most burdensome malignancies worldwide, with incidence and mortality projected to remain high in the coming decades [1–7]. NSCLC accounts for the vast majority of cases [8] and, in advanced stages, management focuses on prolonging survival while maintaining quality of life (QoL) [9, 10]. In this single-center cohort, patients who quit smoking at diagnosis achieved better disease control, experienced fewer toxicities – particularly less neutropenia – and had longer OS (11 versus 7 months) than those who continued to smoke. These findings align with previous evidence showing that post-diagnosis cessation favourably influences outcomes in lung cancer [11, 12].

Our survival data are consistent with prior studies. Parsons *et al* [11] reported significantly higher all-cause mortality in patients who continued smoking after an early-stage lung cancer diagnosis, while Koshiaris *et al* [12] demonstrated longer survival in those who stopped smoking within the first year. Although our cohort included patients with locally advanced and metastatic disease, the observed benefit of cessation suggests that its positive impact extends across disease stages, supporting systematic cessation counseling at diagnosis.

### Smoking cessation during treatment

An important clinical observation in our cohort was that several patients who were active smokers at diagnosis successfully quit smoking during the course of treatment. Clinically, smoking cessation during therapy appeared to be associated with improved treatment tolerability, including fewer treatment-related toxicities and less frequent treatment interruptions. This reinforces the value of continuous, individualised smoking cessation support throughout treatment, rather than limiting interventions to the point of diagnosis.

Treatment tolerability was also influenced by smoking status. We found a higher incidence of treatment-related adverse events, driven by haematologic toxicity, among active smokers. This parallels findings from prospective studies in unresectable stage III NSCLC, where continued smoking correlated with increased rates of treatment-related esophagitis [13]. Possible mechanisms include smoking-induced bone marrow suppression, systemic inflammation, altered drug metabolism and tissue hypoxia, which may collectively increase chemotherapy toxicity and reduce dose intensity.

QoL findings from the literature reinforce these results. Repeated QoL assessment is a validated outcome in advanced NSCLC [14, 15], and multiple studies show that active smokers have lower QoL scores than ex- or never-smokers [16–19]. Smoking cessation is associated with significant improvements in physical and emotional functioning [20, 21]. Given the link between QoL, treatment adherence and survival, these improvements may partially explain the survival advantage observed in our study.

Socioeconomic factors also play a role. Individuals with lower socioeconomic status are less likely to quit smoking and may have reduced access to cessation resources [22]. Addressing these disparities – through free pharmacotherapy, behavioural counseling and integration of cessation services into oncology care – is critical, particularly in resource-limited settings.

The distribution of histologies and high proportion of metastatic presentations in our series are consistent with global data from the IASLC staging project and GLOBOCAN estimates [23, 24]. Because tobacco exposure is closely linked with certain histologies, especially squamous cell carcinoma, targeted cessation strategies may be particularly relevant in these subgroups.

### Clinical implications

Our results support:

- systematic assessment of smoking status at every visit;
- immediate, opt-out referral to cessation programs at diagnosis;
- provision of evidence-based pharmacotherapy (nicotine replacement, varenicline, bupropion) combined with behavioural counseling;and
- integration of cessation support into multidisciplinary care pathways. Embedding cessation within treatment planning could reducetoxicity, preserve dose intensity and improve both survival and QoL. Continuous support is particularly important because somepatients may quit during therapy and these individuals appear to benefit in terms of treatment tolerability.

## Limitations

The retrospective, single-center nature of our study introduces potential confounding and selection bias. Smoking status was based on medical records and may be subject to misclassification, and detailed smoking exposure data (e.g., pack-years, biochemical verification) were unavailable. Additionally, some patients who were active smokers at diagnosis quit during treatment, which may introduce variability in treatment outcomes. Follow-up was relatively short. Nevertheless, the consistency of findings across treatment response, toxicity and survival, along with alignment with prior research, supports the robustness of our conclusion.

## Conclusion

Smoking cessation at diagnosis is associated with improved survival, reduced treatment-related toxicity and potentially better QoL in patients with advanced NSCLC. Notably, some patients who were active smokers at diagnosis successfully quit during treatment, which appeared to improve treatment tolerability. These results reinforce prior evidence that quitting smoking after a lung cancer diagnosis significantly prolongs survival [11, 12] and reduces treatment-related adverse events [13]. Structured cessation support should be integrated into standard oncology care to optimise patient outcomes. Prospective multicenter studies are warranted to confirm these findings and determine the most effective cessation strategies.

## Conflicts of interest

The authors declare that they have no conflicts of interest.

## Funding

No funding was received for this study.

## Figures and Tables

**Table 1.** Tumour response following first -line chemotherapy according to smoking status.

Response type	Total (n = 100)	Quit smoking (n = 60)	Continued smoking (n = 40)
Complete response	3 (3%)	2 (3.3%)	1 (2.6%)
Partial response	26 (26%)	18 (29.5%)	8 (20.5%)
Stable disease	44 (44%)	28 (45.9%)	16 (41.0%)
Progressive disease	27 (27%)	13 (21.3%)	14 (35.9%)

## References

[1] Sharma R (2022). Mapping of global, regional and national incidence, mortality and mortality-to-incidence ratio of lung cancer in 2020 and 2050. Int J Clin Oncol.

[2] National Cancer Institute (2025). SEER Cancer Stat Facts: Lung and Bronchus Cancer.

[3] Doll R, Hill AB (1954). The mortality of doctors in relation to their smoking habits. BMJ.

[4] Terry LL (1964). (Washington, DC: US Department of Health, Education, and Welfare). Smoking and Health: Report of the Advisory Committee to the Surgeon General of the Public Health Service.

[5] Deng Y, Zhao P, Zhou L (2020). Epidemiological trends of tracheal, bronchus, and lung cancer: a population-based study. J Hematol Oncol.

[6] American Cancer Society (2021). The Global Economic Cost of Cancer.

[7] Kutikova L, Bowman L, Chang S (2005). The economic burden of lung cancer and the associated costs of treatment failure in the United States. Lung Cancer.

[8] Gridelli C, Rossi A, Carbone DP (2015). Non-small-cell lung cancer. Nat Rev Dis Primers.

[9] Legodec J, Berard H (2013). L’analyse de la qualité de vie en oncologie thoracique. Rev Mal Respir Actual.

[10] Underner M, Perriot J, Peiffer G (2014). Influence du tabagisme sur la qualité de vie des patients atteints de cancer bronchique. Rev Mal Respir.

[11] Parsons A, Daley A, Begh R (2010). Influence of smoking cessation after diagnosis of early stage lung cancer on prognosis: systematic review of observational studies with meta-analysis. BMJ.

[12] Koshiaris C, Aveyard P, Oke J (2017). Smoking cessation and survival in lung, upper aero-digestive tract and bladder cancer: cohort study. Br J Cancer.

[13] Cox LS, Africano NL, Tercyak KP (2002). Smoking behavior of patients with stage IIIA/IIIB non-small cell lung cancer. Lung Cancer.

[14] Hollen PJ, Gralla RJ, Rittenberg CN (2004). Quality of life as a clinical trial endpoint: determining the appropriate interval for repeated assessments in patients with advanced lung cancer. Support Care Cancer.

[15] Cella D (2003). Impact of gefitinib on non-small cell lung cancer-related symptoms as measured by the Functional Assessment of Cancer Therapy-Lung scale. Semin Oncol.

[16] Hays JT, Croghan IT, Baker CL (2012). Changes in health-related quality of life with smoking cessation treatment. Eur J Public Health.

[17] Wilson D, Parsons J, Wakefield M (1999). The health-related quality-of-life of never smokers, ex-smokers, and light, moderate, and heavy smokers. Prev Med.

[18] Hillmann M, Silcock J (1997). A comparison of smokers’ and ex-smokers’ health-related quality of life. J Public Health Med.

[19] Martinez JAB, Mota GA, Vianna ESO (2004). Impaired quality of life of healthy young smokers. Chest.

[20] Shaw JW, Coons SJ, Foster SA (2001). Responsiveness of the smoking cessation quality of life (SCQoL) questionnaire. Clin Ther.

[21] Stewart AL, King AC, Killen JD (1995). Does smoking cessation improve health-related quality of life?. Ann Behav Med.

[22] Hiscock R, Bauld L, Amos A (2012). Socioeconomic status and smoking: a review. Ann N Y Acad Sci.

[23] Chansky K, Detterbeck FC, Nicholson AG (2017). The IASLC Lung Cancer Staging Project: external validation of the revision of the TNM stage groupings in the eighth edition of the TNM classification of lung cancer. J Thorac Oncol.

[24] Bray F, Ferlay J, Soerjomataram I (2018). Global cancer statistics 2018: GLOBOCAN estimates of incidence and mortality worldwide for 36 cancers in 185 countries. CA Cancer J Clin.

